# Supplementary material for: Are carbon emissions trading and green financial instruments synergistic? -Comprehensive quantitative research based on content analysis
Source: PLoS One. 2024 Mar 7;19(3):e0298601. doi: 10.1371/journal.pone.0298601 (PMC10919601; doi:10.1371/journal.pone.0298601)
Supplement: S1 Data — (ZIP) [file pone.0298601.s001.zip › Data/地级市Z_F工作报告.zip!/╟α║ú/╬≈─■╩╨/└┤╫╘╣1⁄2╓┌║┼í╛┬φ┐╦╩2╛▌═°í┐_2019.docx]

各位代表：

现在，我代表市人民政府向大会报告工作，请予审议，并请政协委员提出意见。

一、回顾2018，绿色发展、幸福西宁迈出新步伐

过去一年，面对复杂严峻的宏观环境和压力叠加的多重挑战，在省委省政府和市委的坚强领导下，我们始终坚持以习近平新时代中国特色社会主义思想为指导，全面贯彻党的十九大和十九届一中、二中、三中全会精神，牢固树立“四个意识”，不断坚定“四个自信”，坚决做到“两个维护”，以“两个绝对”为标准，以“两个绝对”具体化为载体，全面落实省第十三次党代会和省委十三届三次、四次全会、市第十四次党代会和市委十四届六次、七次全会部署，紧紧围绕全面落实“四个扎扎实实”重大要求、深入实施“五四战略”、奋力推进“一优两高”，统筹做好稳增长、促改革、调结构、惠民生、防风险各项工作，较好完成了市十六届人大四次会议确定的各项目标任务，绿色发展样板城市和新时代幸福西宁建设取得了新进展。

2018年，是奋发前行重作为，经济运行稳中有进的一年。我们积极应对经济运行中出现的新情况新问题，逐月分析研判经济形势，严格执行“710”工作法，精心开展“百日攻坚”“会战黄金季”等四大专项行动和“四个奔着去”大抓落实活动。经济发展势头良好。实现地区生产总值1286.4亿元、增长9%，全社会固定资产投资增长9%，社会消费品零售总额564.4亿元、增长6.7%，地方公共财政预算收入92.9亿元、增长17.4%，城乡居民人均可支配收入分别达到32500元、11504元，分别增长8.2%、9.1%，多项指标增速高于全省。新兴产业全面提速。实施工业新兴产业倍增计划，启动建设光热产业园，开工国电投N型电池全国首个光伏智能工厂，亚洲硅业等5家企业获批全国“绿色工厂”。北捷隔膜、变形镁合金等122个新兴产业项目顺利推进，新能源、新材料产业增加值分别增长62.9%和25.7%，高技术产业增加值增长32.8%，占规模以上工业的18.6%。现代服务业提档升级。第三产业占比首次突破60%，对经济增长的贡献率达50.3%，成为拉动经济增长新引擎。健康养老、物流快递、网络消费等新业态蓬勃发展，电子商务交易额增长25%。海洋馆、熊猫馆、冰球馆等高端旅游项目加速推进，建成日月山等8条乡村旅游示范带，首届青海地方特色小吃大赛暨西宁美食节广受赞誉，全市旅游人数和收入分别达到2460.7万人（次）、312.4亿元，增长15.1%和24.5%，成为全国游客数量增长最快的十大“网红城市”。农业生产保持稳定。一产增加值增长4.2%。蔬菜、粮、油产量分别达到68.5万吨、22.6万吨、7.1万吨，特色农作物种植面积比重提高到76.5%，新认定老爷山、云谷川等4个省级现代农业产业园。

2018年，是绿色发展筑本底，生态环境持续改善的一年。我们深刻认识“生态似水、发展如舟”，积极推进“高原绿”“西宁蓝”“河湖清”建设行动，展开了天水一色、绿意盎然、鸟语花香的生态画卷。全城绿化量质并举。三北、天保等重点工程造林59.8万亩，提前两年完成南北山三期造林任务。园博园、西堡绿芯森林公园景观栽植2030亩、造林3.3万亩。建设街头绿地39处，绿化道路27条，新增园林绿地1275亩。湟水国家湿地公园正式命名，建成开放的北川湿地等4个公园游园成为市民休闲好去处。全国大规模国土绿化现场会在西宁召开并推广我市经验。大气质量显著改善。建立网格化大气环境监管体系，落实建筑工地文明施工“10个100%”，汽车尾气治理、冬季供暖清洁能源改造走在全国前列，空气质量连续4年位居西北省会城市第一。水域整治力度空前。健全四级复合型河湖长制管理体系，成功创建全国水生态文明城市。建成南川河水生态文明（一期）、大华水库等重点水利工程，综合治理155条小流域。10万吨规模的第六污水处理厂建成投运，四污改扩建达标排放，小峡桥国控断面水质达到IV类标准。环保整改落地见效。中央环保督察反馈问题已整改6项，剩余8项正在有序推进。完成祁连山水泥有限公司矿区和12处历史遗留矿山生态恢复治理，绿色矿山示范创建卓有成效。

2018年，是规建并举增颜值，城乡面貌大幅提升的一年。我们坚持以城带乡、区域协调，有序推进新型城镇化和城乡一体化，加快城乡融合发展。城市建设再掀热潮。主动融入兰西城市群，谋划启动“大西宁”规划建设，开展空间规划“多规合一”试点。实施火烧沟、苦水沟等“城市双修”示范项目。建成地下综合管廊37公里，完成21.6平方公里海绵城市试点区建设。实施多巴“四河一湖十一条沟”治理，新华联国际旅游城、市民中心已具雏形。城市治理更加精细。城市主干道机械化清扫、清洗作业实现全覆盖。全面推行街长制，建立城市管理市民监督平台。生活垃圾分类示范区居民覆盖率达17%，走在西北省会城市前列。县域基础不断夯实。制定“1+10+1”行动框架，编制完成乡村振兴战略实施规划。建设“四好农村路”462公里，改造农村电网830公里。开工2个建制镇污水处理厂和5个乡镇生活垃圾填埋场，新（改）建农村卫生厕所5087座。完成91个村风貌提升工程，5个美丽城镇、79个高原美丽乡村绽放新姿。

2018年，是改革开放显活力，发展后劲明显增强的一年。我们着力破解制约发展的体制机制障碍，加快打造改革开放新高地。重点改革亮点纷呈。聚焦“去降补”，关停出清思维铜业等落后企业，商品住房去化周期缩短为5个月，实现上下游产业对接金额215亿元，减免小微企业增值税、所得税6.1亿元。深化“放管服”改革，公布375项最多跑一次事项，取消197项涉及基层群众办事创业证明事项，出台优化营商环境“1+8”政策措施，成为营商环境上升最快TOP10城市。建成全省首家城市运行指挥中心、城市级大数据基础平台，荣获2018年中国领军智慧城市称号。深化四级紧密型医联体改革，“从医联体迈向健康服务共同体”列为全国推进医改、服务百姓健康十大新举措，家庭医生和健康承包重点人群签约率87.7%。入选全国居家和社区养老服务改革试点优秀示范城市，农村养老、医养融合双证经营、民族特色养老模式走在全国前列。对外开放成果丰硕。西宁综保区上报国务院审批，西宁机场升级为国际机场。设立15个特色商品国际营销中心、13个进口商品展销平台，外贸进出口总额占全省68%。集中开展春秋季招商、邀请客商进西宁等活动，依托城洽会、世界新能源大会等平台，深化“双50”重点项目招商，卓丰汽车用铝板、多巴自驾车营地等33个项目落地开工，招商引资省外到位资金269亿元。创新能力持续攀升。青海国家高新区正式筹建，西宁开发区评为国家级创新创业特色载体基地。全市高新技术企业和省级科技型企业分别占全省81.9%和73.3%。专利申请量3313件，授权量1939件，占全省比重均超过70%。

2018年，是改善民生添福祉，群众得到更多实惠的一年。我们在改善民生上投入更大财力精力，教育、科技、食药监管等惠民领域资金投入分别增长13.5%、50.2%和22.3%。民生保障扎实有力。城镇新增就业3.2万人，农村劳动力转移就业38.9万人（次），城镇登记失业率控制在2.45%。创建4家国家级放心肉菜示范超市，打造11条食品安全示范街、160家示范企业和示范店，6700余家餐饮服务单位实行分类定级店外挂牌公示。新建13个社区老年日间照料中心和50个农村老年之家，5.8万人（次）享受政府购买养老服务。改造棚户区12712套，分配公租房2300套，综合改造老旧楼院7653套。加大城乡困难群众精准帮扶力度，发放社会救助金3.9亿元，累计全额资助1528名贫困大学生。严控21种蔬菜批零差率，妥善应对“非洲猪瘟”疫情，蔬菜和猪肉价格分别低于全国36个大中城市均价4.2和13.7个百分点，平稳理顺天然气和集中供暖价格，居民消费价格指数累计上涨2.7%。社会事业全面发展。实施教育布局调整等8大类93个项目，基本消除义务教育超大班额，普惠性幼儿园占比达80%。市二医院儿童诊疗中心、湟源县中医院投用，新增39所基层中藏医馆，市第一医疗集团荣获全国“改善医疗服务优秀医院”称号。新（改）建162个基层综合性文化服务中心，沈那遗址保护规划获国家批准，推出《绿水青山·幸福西宁》等一批文艺精品，《尕布龙》在全国巡演。体育健身器材实现村、社区全覆盖，在全省率先建成“10分钟体育健身圈”，射击名将纪晓晶获亚运会金牌。加大社会管控和源头治理，推进社会治安分级评价改革，城市公共安全指数位居全国第二。深入开展安全生产重点领域专项整治，连续8年评为全省安全生产先进地区。深化民族团结进步创建，依法管理宗教事务，民族和睦、宗教和顺、社会和谐的良好局面不断巩固。

各位代表，我们积极回应群众关注关切、切实解决市民所需所盼，在市十六届人大四次会议上向全市人民承诺的十大类35件民生实事全部完成！

2018年，是履职尽责提效能，自身建设不断强化的一年。我们坚决落实市委各项工作部署，坚持重大事项向市委请示报告，重大问题集体研究。认真执行人大及其常委会决议决定，主动接受人大工作监督、法律监督，自觉接受政协民主监督，办理答复人大代表建议140件、政协委员提案142件，办复率和满意率均为100%。严格落实全面从严治党各项制度，自觉执行党风廉政建设责任制，强力整治形式主义、官僚主义，行政效能和服务水平进一步提升。法治政府建设在中国法治政府评估报告中排名上升9位，制定颁布了全国首部绿色发展样板城市促进条例。广泛听取民主党派、工商联、无党派人士意见建议，国防动员、双拥、普法、新闻广电出版、外事侨务、信访、人防、地震、气象、供销合作、工青妇、慈善、残疾人、红十字等事业健康发展。

一年来，全市237万各族干部群众敢于担当有干劲、举力攻坚有成效，描绘了打造绿色发展样板城市的新画卷，掀开了建设新时代幸福西宁的新篇章！我们全面落实省委省政府“做强西宁、服务全省”“打造宜居宜业大西宁”重大要求，积极应对持续加大的经济下行压力，增速继续位居全国省会城市前列，对全省的贡献率超过60%。我们迎接了中央脱贫攻坚专项巡视和国务院第五次大督查，如期实现1.45万贫困人口脱贫、150个贫困村退出、三县达到“摘帽”标准的目标，全面完成三年攻坚任务。“放管服”改革“西宁样本”受到国务院通报表扬。我们牢牢把握创新驱动和动力转换核心关键，突破微孔铜箔研发技术并实现量产，光伏产业技术中心成为国际国内代表性实验室，单晶硅电池平均转换效率达到国际先进水平。中国联通三江源大数据中心、比亚迪动力电池等一批重大项目建成投产，千亿元锂电产业基地、光伏制造中心影响力和辐射力明显提升。我们全面完成“畅通西宁”三年攻坚行动和“美丽夏都·清洁西宁”品牌建设收官战，建成凤凰山路等101个项目，人均道路面积提高至12.3平方米，交通拥堵指数下降41.6%，再次入选全国十大缓解拥堵城市。400辆新能源出租车投运，主城区公交扫码乘坐全覆盖，市民出行更加便捷高效。开展“城市清洗”活动，下大力气净化城市“立面空间”，洁净优美的城市环境成为“西宁形象”的靓丽名片。

各位代表，改革开放40年，西宁从西部边陲小城蜕变为青藏高原和丝绸之路经济带上的一颗璀璨明珠，40年的攻坚克难和风雨砥砺换来今天的破茧成蝶。这40年，我们紧抓国家推进西部大开发、“一带一路”建设、脱贫攻坚、乡村振兴等政策机遇，按照省委省政府决策部署，在市委的坚强领导下，坚持一张蓝图绘到底、一茬接着一茬干，锲而不舍抓发展、竭尽全力保民生、坚定不移促改革，各族干部群众和各级各部门众志成城、接续奋斗，各民主党派监督支持、倾心参与，社会各界鼎力协作、关心帮助，在困难和压力面前心往一处想、劲往一处使、拧成一股绳，以昂扬向上的精神状态破解一道道难题、攻克一道道难关，凝聚了“干在实处、走在前列”的精气神。在此，我代表市人民政府，向全市各族干部群众，向人大代表、政协委员、各民主党派、工商联、无党派人士和各人民团体，向驻宁部队指战员、武警官兵、公安干警，向所有关心、支持西宁建设发展的同志们、朋友们，致以崇高的敬意和衷心的感谢！

在肯定成绩的同时，我们也清醒地看到，全市经济社会发展仍面临不少困难和问题，主要表现为：发展质量和效益还不高，企业生产经营困难增多；科技创新能力不足，高新技术产业占比低、带动力不强；资源环境约束趋紧，节约资源和保护环境任务艰巨；基础设施、民生改善、社会事业、社会治理等领域还存在不少短板和薄弱环节，供给与需求的矛盾依然突出；干部作风还需持续改进，行政效能有待进一步提高。我们将高度重视，认真对待，下大力气解决。

二、展望2019，紧盯目标、加压实干开创新局面

今年是中华人民共和国成立70周年，是全面建成小康社会的关键之年，也是贯彻落实省委“一优两高”战略部署、“打造宜居宜业大西宁”迈开新步伐展现新作为的关键之年，更是打造绿色发展样板城市、建设新时代幸福西宁滚石上山、爬坡过坎的关键之年，做好政府各项工作至关重要。

今年政府工作的总体要求是：坚持以习近平新时代中国特色社会主义思想为指导，全面贯彻党的十九大和十九届一中、二中、三中全会及庆祝改革开放40周年大会、中央政治局民主生活会、中央经济工作会议、十九届中央纪委三次全会、中央政法工作会议精神，牢固树立“四个意识”，不断坚定“四个自信”，坚决做到“两个维护”，以“两个绝对”为标准，以“两个绝对”具体化为载体，坚持和加强党的全面领导，统筹推进“五位一体”总体布局，协调推进“四个全面”战略布局，坚持稳中求进工作总基调，坚持新发展理念，坚持推动高质量发展，坚持以供给侧结构性改革为主线，坚持深化市场化改革、扩大高水平开放，全面落实省第十三次党代会、省委十三届五次全会、市第十四次党代会及市委十四届八次全会部署和建军书记、刘宁省长调研西宁工作时讲话要求，围绕全面落实“四个扎扎实实”重大要求、深入实施“五四战略”、奋力推进“一优两高”，坚持生态优先、绿色发展，统筹推进稳增长、促改革、调结构、惠民生、防风险工作，保持经济运行在合理区间，进一步稳就业、稳金融、稳外贸、稳外资、稳投资、稳预期，建设现代产业体系，打好三大攻坚战，提升社会治理水平，落实新时代党的建设总要求，为全面建成小康社会打下决定性基础，奋力开创绿色发展样板城市、新时代幸福西宁建设新局面，全力当好新青海建设的“领头雁”、“一优两高”的排头兵、大美青海的“施工队”，以优异成绩庆祝中华人民共和国成立70周年。

按照市委对今年工作的总体要求和决策部署，建议今年经济社会发展的主要预期目标是：全市地区生产总值增长7.5—8%；固定资产投资增长8%；社会消费品零售总额增长8%；地方公共财政预算收入增长7.5%；全体居民人均可支配收入持续高于经济增速；城镇登记失业率控制在2.5%以内；城市空气质量优良率、居民消费价格指数、节能减排完成省定目标。

各位代表，以上预期目标，一是通盘考虑复杂多变的经济形势和持续加大的下行压力，保持我市经济平稳运行的不确定因素增多，长期支撑高速增长的外部环境有所变化；二是符合推动经济由高速度增长向高质量发展要求，与现阶段增长潜力相适应，着力为“坚持生态保护优先”“推动高质量发展”留出一定回旋空间；三是与全面建成小康社会和“十三五”规划目标相衔接，在目标设定上适当高于国家和全省，保持相对较高的增长速度，体现“做强西宁、服务全省”的要求。

各位代表，为全省经济增长多做贡献是西宁的责任，提高经济发展质量效益、为全省经济结构优化多做贡献是西宁的担当，完成今年目标任务，压力不小、困难很大。我们必须矢志不渝坚持党的全面领导，把推进“两个绝对”具体化作为牢固树立“四个意识”、坚决做到“两个维护”的思想方法和实践载体，确保各项事业始终沿着正确方向前进。我们必须坚持生态优先、发展率先，紧紧抓住绿色发展新机遇，坚定走生产发展、生活富裕、生态良好的绿色发展道路，让绿水青山变成金山银山。我们必须深入践行以人民为中心的发展思想，坚持履职为民的坚定立场和价值取向，带领和依靠群众一步一个脚印地向胜利迈进。我们必须以“干在实处、走在前列”的精神面貌，勇挑重担、甘于奉献，发挥示范引领作用，努力争取更好成绩，为全省人民的幸福安康做出新贡献！

三、聚焦“一优两高”战略部署，奋力打造绿色发展样板城市

深入贯彻新发展理念，坚持以生态保护优先为统领，以深化供给侧结构性改革为主线，坚定不移打好三大攻坚战，加速推动产业转型升级，深化改革扩大开放，在更高层次上推动绿色发展。

（一）着力打好三大攻坚战，夯实高质量发展基础。以攻坚必胜的信心，坚决打好防范化解重大风险、精准脱贫、污染防治三大战役，全力跨越决战决胜全面建成小康社会的“关口”。

打好防范化解重大风险攻坚战。做好政府和企业债务风险的跟踪评估和防范化解工作，加快债务有序偿还，确保总量不增加、存量逐年减、底线不突破。把降低企业杠杆率作为重中之重，促进企业盘活存量资产，推进资产证券化，支持市场化法治化债转股，加大股权融资力度，强化企业特别是国有企业财务杠杆约束，确保资产负债率处在可控范围。深入开展涉金融领域失信问题专项治理，做好防范和处置非法集资工作，牢牢守住不发生区域性系统性金融风险的底线。坚持“房住不炒”定位，合理引导房地产市场预期和购房行为，确保房价总体平稳。

打好精准脱贫攻坚战。抓好中央脱贫攻坚专项巡视交办问题整改。落实消费扶贫、生态扶贫等各项扶贫政策，做好南京对口帮扶和扶智扶志工作。开展摘帽县、退出村、脱贫户“回头看”，巩固脱贫成果后续扶持措施，切实做到脱贫不脱帮扶、脱贫不脱政策，决不让一个贫困群众在全面小康中掉队。探索多种模式发展村集体产业，实现村级集体经济收入稳定增长。探索建立特殊类型贫困家庭长效脱贫解困机制，密切关注收入水平略高于建档立卡贫困户群体的生产生活，坚决防止出现返贫和新生贫困。

打好污染防治攻坚战。紧盯中央环保督察问题整改，全力以赴打好污染防治八场标志性战役，严控问题增量，决不把污染的空气、污浊的水体、污损的土壤带进全面小康社会。强化大气污染联防联控，编制大气污染颗粒物源清单和限期达标规划，建成市区监管平台和100个智慧化建筑工地，淘汰县城10蒸吨以下燃煤锅炉，确保空气质量优良率继续走在西北省会城市前列。启动西川河生态综合整治，实施城镇污水处理“提质增效”三年行动，基本完成市区雨污分流工程，推动污水管网向村庄延伸。加快铬渣等重金属污染场地修复治理，巩固重金属污染防治成果，在册矿山全部建成绿色矿山。

（二）着力强化生态建设和环境治理，筑牢绿色发展本底。尊重自然、热爱自然、保护自然是西宁人民永恒不变的绿色追求，我们将持之不懈加强生态文明建设，让西宁的天更蓝、水更清、地更绿。

筑牢生态屏障。围绕国土绿化提速行动，把国家湟水规模化林场作为构建森林生态系统的重要支撑，以三县规模化造林为重点，探索森林资源经营管理、投融资机制等创新试点，完成造林绿化53.5万亩，森林覆盖率提高到34.5%。建设园博园2000亩景观林带，完成水利灌溉、游览设施等配套工程。创建“义务植树+互联网”试点城市，建设国家级全民义务植树基地。

打造高原水城。坚持“治宁方略，水为大政”，编制全市水网总体规划，协调推进“引黄济宁”工程，实施石—南水库水系连通、南川河水生态文明（二期）等重点项目，构建多元互补、调控自如的“大西宁”都市圈水网体系。开展水资源消耗总量和强度双控行动，建设节水型城市。落实“一河（湖）一策”，建立河湖长制综合管理信息平台，综合治理黑林河、马圈沟等33条小流域。加强沿湟水干支流生态治理保护、湿地修复，为群众提供水清、岸绿、景美的水生态环境。

倡导绿色生活。健全固废收运处置信息化监管体系，一般工业固废处置利用率达到95%以上。开工垃圾焚烧发电项目，创建绿色单位、绿色家庭，生活垃圾分类达标小区覆盖率达到60%。在基础设施、房地产开发等领域推广绿色建筑建材和装配式建筑。新增充电桩500个，公交车、出租车电动化率分别达到30%和10%，让绿色出行成为群众的最佳选择。

（三）着力推动制造业高质量发展，构建现代产业体系。聚焦“锂电、光伏光热、有色合金高新材料、特色化工、生物医药和高原动植物资源精深加工”五大产业集群，坚持创新驱动发展，促进工业化与信息化深度融合，加速壮大提升新兴增长极。

做强高新技术产业。围绕建设国家能源革命示范省，培育壮大新能源、新材料、生物医药等战略性新兴产业，加速释放新产能、新动能。光伏光热抓住全省特高压外送通道和电源电站建设机遇，鼓励现有企业进一步兼并重组、扩大产能，加快建设光热产业园，再引进培育一批光热发电龙头企业。锂电完善隔膜、电解液和电池检测回收等配套产业，实施比亚迪二期、时代新能源扩产等引领性项目，夯实千亿锂电产业基础，成为全国重要的锂电产业基地。瞄准新能源汽车整车制造，加快打造变速箱、汽车铝板材等轻量化车用零部件后续产业链。高新材料发挥铝镁合金高新材料产业园集聚作用，推动青镁变形镁合金、欧莱德铝型材、海通电缆等项目年内投产，开工中利光纤拉丝、中复神鹰碳纤维等补链延链项目。生物医药和高原动植物资源精深加工加快重大疾病治疗药物创新和产业化，实施央宗药业地黄梓醇片、国肽生物肽提取加工等项目，研发具有较高科技含量、无污染的系列食品和保健品。

改造提升传统产业。坚持新旧并重、旧中求新，运用新技术、新模式优化升级传统产业，让“老树发新芽”。特色化工推动大美煤业烯烃项目尽快投产，延伸发展乙烯、丙烯产业链；开发高端精细耗氯产品及高性能合成树脂，延长氯碱产业链，打造千亿特色化工产业集群。有色金属冶炼及加工实施广泰铸造铝合金、盐湖镁板带等重点项目，扩大工业铝型材、镁合金结构材料等中下游产品生产规模。装备制造促进大数据、人工智能在企业研发设计、生产制造的全流程运用，建设聚能电力等一批智能工厂和数字化车间。藏毯绒纺加快优势企业兼并重组，建设检测认证、展销中心等服务平台，塑造区域和国际品牌。

培育发展数字经济。坚持产业数字化、数字产业化，培育发展大数据产业。依托市级大数据基础平台，谋划实施“一带一路”国际贸易信息港、高原生态大数据实验室等重点工程，建设绿色发展生态链数字化监控平台。加快“宽带中国”示范城市、电信普遍服务建设试点，推进网络提速降费，提前布局5G等新一代无线网络。促进实体经济和数字经济、工业化和信息化深度融合，形成数据驱动、跨界融合、开放协同的数字经济生态和支撑体系，深化数字技术在交通、医疗、环保等领域的广泛应用，打造青藏高原大数据产业集聚区。

强化科技创新支撑。深化创新型城市建设，引导和支持创新要素向企业集聚，促进科技成果向现实生产力转化，打造全省科技创新中心。推动高新区与大学园区融合发展，探索设立产学研经济联合体，开工建设科技城，运行西宁科技大市场，打造全省科技创新新地标。规划建设人工智能产业集聚区，强化人工智能前瞻布局。启动建设国家车辆产品高原质量监督检验中心。实施“百项创新攻坚”工程，建设铝镁合金高新材料产业研发中心等5大科技创新平台，重点攻克新型薄膜电池、盐湖提锂、航空航天高性能用铝等引领技术，助推主导产业提质转型。实施西宁市“引才聚才555计划”，突出“项目揽才、产业聚才”，完善柔性引才引智机制，吸引各类创新人才加速集聚。

发挥园区引领作用。加快产城融合，促进园区与城区规划衔接、设施联通、功能互补，提升区域经济整体实力。推动园区集约高效发展，优化提升各园区产业布局定位，制定单位土地投入和产出强度标准，追求“亩产效益”，加快腾退污染、低效、粗放型产业用地，引进一批智能制造、研发服务、大数据等高新技术项目。立足三县特色资源禀赋，加快建设青藏高原原产地绿色食品、铝镁合金高新材料、鲁沙尔民族文化旅游产业园，补齐县域经济发展短板。瞄准“中国制造2025”，扶持龙头企业做大做强，努力在光伏、汽车动力电池、铜箔和光纤制造等领域打造若干个国家级“单项冠军”。深化开发区循环化改造示范和低碳工业园区试点，开展重点行业能效“领跑者”行动，规模以上工业单位增加值能耗下降2.3%。

（四）着力扩大有效投资促进消费，强化绿色发展动力支撑。坚持以结构调整增加绿色投资、以消费提档升级培育绿色发展新动能，持续释放需求潜力，双管齐下推动高质量发展。

切实稳定有效投资。聚焦国家确定的生态环保、脱贫攻坚、基础设施等十大补短板领域，发挥投资的关键拉动作用，深化“绿色项目生成年”活动，提高投资的精准性和有效性。持续优化投资结构。围绕构建现代产业体系，加大锂电、光伏光热、生物医药等绿色产业投入，为新兴产业加速集聚注入新活力。以“幸福西宁绿色交通”三年行动计划为牵引，加快建设城乡公共服务、水电路等基础设施，增强城市载体服务能力。继续增加环境保护、生态工程、现代农业投入，夯实绿色发展根基。加快补齐教育、医疗、住房、扶贫等领域短板，保障高品质生活需求。狠抓重点项目建设。落实“周检查、旬协调、月督办”推进机制，加大土地、资金等要素保障，推进北山市场改造、元堡子等片区拆迁，集中精力实施130个市级重点项目，确保投资持续健康增长。加速项目谋划生成。对接国家和全省产业政策和资金投向，实施“十三五”中后期绿色发展重大项目规划，围绕兰西城市群、“一优两高”、乡村振兴和数字经济等领域，再谋划储备一批重大项目。抢抓国家大幅增加地方政府专项债机遇，做深做细土储、棚改等项目前期，争取专项资金增长10%。

深入挖掘消费潜力。主动顺应消费需求变化新趋势，丰富提升现代服务业，满足群众多样化消费需求。依托新城吾悦广场、北川万达广场、红星美凯龙爱琴海、国际会展中心等商业综合体，在城市东南西北打造一批新的商贸集聚区、商业新中心。实施水井巷特色美食街区改造，建设“一带一路”特色商品展示馆，把朝阳物流园改造提升为全省高端生活资料配送中心。加快建设电子商务产业园，构建线上线下融合的智慧社区便民服务体系，电子商务交易额达到450亿元。统筹健康、养老、购物、法律咨询等资源，打造“互联网+居家服务”创新平台，发展家庭用品配送、在线教育、网络医生咨询等新业态，让群众足不出户享受便捷多样服务。

大力发展全域旅游。促进旅游与文化产业、城市功能与旅游功能融合发展，努力将西宁建设成国际旅游名城、中国西部区域旅游集散中心和青藏高原特色旅游服务基地。以中国西部自驾车旅游联盟为龙头，打造国道227线等3条生态旅游观光风景道，新建4处自驾车营地、2个旅游集散中心，办好第四届西部自驾游联盟年会、第二届青海地方特色小吃大赛暨西宁美食节等活动，扩大西宁旅游品牌影响力。全面建成10条乡村旅游示范带。新华联国际旅游城和海洋馆、熊猫馆、冰球馆等精品项目向市民开放。丰富鲁沙尔民族文化旅游产业园品质内涵，建成王牌民族文化旅游区；推动丹噶尔古城保护与开发，打造历史文化名镇；依托藏医药文化博物馆等平台，申报青海博物馆群5A级景区。

（五）着力推动乡村振兴，促进城乡融合发展。坚持农业农村优先发展，加快基础设施和公共资源向农村延伸，推动农业全面升级、农村全面进步、农民全面发展。

深化农业供给侧改革。坚持质量兴农、绿色兴农，严守耕地保护红线，清理整治“大棚房”，坚决遏制农地非农化，持续提升耕地质量。稳定粮食生产，加大农业“两区”建设保护力度，粮食生产功能区保持在37万亩。围绕“一线四川”现代设施农业，优化农业生产力布局，大通双新公路沿线建设绿色循环优质蔬菜产业基地，在湟中西纳川、大通景阳川、城中总寨塬等建立一批特色高效蔬菜生产区和标准园，提高本地蔬菜供给能力。发展特色生态畜牧业，建设奶牛、生猪、家禽标准化规模养殖区，保证肉禽蛋奶等“菜篮子”产品供应充足。深化“清洁田园”行动，确保农药、化肥使用减量20%，创建大通、湟源农产品质量安全县。推进品牌强农战略，探索建立农业产业联盟和农商联盟，打造2个区域公用品牌、30个产品品牌。鼓励农业适度规模经营，建设100个家庭农牧场、10个生态牧场，培育200个农民合作社，促进农村劳动力就近就业创业、增加收入。

加快改善农村人居环境。继续推进农村人居环境整治三年行动，开工建设80个高原美丽乡村，新（改）建农村卫生厕所4000座，提升西塔、宁大等6条公路沿线村庄风貌。强化农村垃圾、污水集中处置，实施大通城关污水处理站、湟中田家寨垃圾填埋场等7个治理项目，建设三县乡镇生活垃圾中转站，90%以上行政村实现垃圾全收集、全处理。

狠抓农村基础设施建设。推动城乡基础设施互联互通、公共服务向农村延伸、社会事业向农村覆盖，新（改）建农村公路180公里，改造农村电网733公里，启动惠及3万群众的农村饮水安全巩固提升工程。扩大县城天然气管网覆盖面，天然气通乡镇率达到30%。深化城乡义务教育集团化办学，实施农村学校厕所、运动场地改造等“3个1000万”项目。新（改）建115个村卫生室，标准化改造比例达到90%。

（六）着力深化改革扩大开放，增创高质量发展新优势。坚定不移将改革开放进行到底，在永无止境的思想解放和实践发展中，一棒接着一棒跑出我们这一代西宁人的好成绩。

落实重点改革任务。瞄准各领域发展不平衡不充分的问题，从群众最关心最直接最现实的利益问题入手，持续推进重点领域和关键环节改革。深化供给侧结构性改革，确保在“巩固增强提升畅通”上取得新成效。加大“放管服”改革力度，建设“一网通办”平台，探索推进“一网一门一次”“不见面”、全程代办等服务模式，让群众办事更加方便舒心。完成机构改革任务，构建优化协同高效的机构职能体系。推进混合所有制改革，开展国有资本投资、运营公司和落实董事会职权改革试点。深化财税体制改革，优化市县财政分配关系，落实好个人所得税等税改政策。继续开展农村土地制度改革三项试点，完成全国农村集体产权制度改革整市推进试点。

全力支持民营经济发展。只有宽阔深水才能养出大鱼，我们将始终坚持“两个毫不动摇”的方针，树立为民营企业主动服务的意识，把民营企业遇到的事当成自己家的事办好，积极构建亲清新型政商关系，以政策活水助力民营经济茁壮成长。落实国家更大规模减税降费政策，加快清理拖欠民营企业账款，坚决破除民营企业发展障碍。继续深化优化营商环境“1+8”政策措施，实施建设项目“多规合一、多评合一、多图联审”，涉企服务效能再提速20%以上。开展企业暖心服务行动，全力解决民营企业在稳定运行、项目建设、产销对接等方面的突出问题。加快转变政府职能，强化政务服务监督管理，大幅减少政府对资源的直接配置，允许更多领域实行独资经营，凡是企业能干的坚决支持企业干，推动更多优质企业脱颖而出、健康发展。

提升金融服务实体经济能力。强化金融办公室职能，建设企业融资之家，为企业提供“一对一”客户经理式服务。用好绿色产业发展基金，引导金融机构对绿色发展项目实行投贷联动。巩固提升“金穗服务惠万家”成果，实施专业合作社金融服务工程，为全市专业合作社提供专门金融支持。发挥市金控、信保集团作用，着力缓解涉农及中小微企业融资难、融资贵等问题。

推动全方位开放合作。借势“一带一路”建设，搭建南向通道运营管理平台，配合推进西宁机场扩建三期和西成铁路等工程，加快形成新丝绸之路青海大通道。启动建设西宁综合保税区，争取设立陆路口岸，将青海丝绸之路国际物流城打造成青藏高原最大的保税物流基地。依托国际会展中心，积极承接国际展会和经贸活动，打造全省对外合作交流的综合性平台。充分利用国际友城关系，加强与中西亚、非洲等地区部分城市的产业对接，努力在矿产、能源、光伏、锂电等领域开展实质合作。推动建立“世界凉爽城市”国际合作机制，高水平参与青洽会、办好城洽会，扩大中国生态环保大会、丝绸之路沿线国际友城峰会影响力。借助外交部青海省全球推介活动、全国工商联民营企业500强峰会等平台，继续开展“双50”重点项目招商，力争引进一批国际国内500强企业，全年招商引资省外到位资金不低于290亿元。

各位代表，“一优两高”战略部署是新时代建设富裕文明和谐美丽新青海的行动纲领，也是西宁打造绿色发展样板城市的主攻方向，我们将扎扎实实把“一优两高”落实到改革发展全领域全过程，努力走出一条具有西宁特色的绿色发展之路！

四、聚焦“打造宜居宜业大西宁”，加快建设新时代幸福西宁

全面落实建军书记、刘宁省长调研西宁工作讲话要求，把规划建设宜居宜业“大西宁”作为城市未来发展方向，把提升在兰西城市群中的极核作用和战略地位作为重点，历史辩证看贡献、立足实际找差距、面向未来绘蓝图，以全球视野找准城市定位，切实扛起省会之责、走好西宁之路，高标准建设美丽幸福新家园。

（一）着力深化城市规律研究，促进区域协调发展。打破眼睛向内的思维定式，放眼世界、面向全国，研究城市未来发展趋势，推进城市从深度开发建设向未来发展转型。

主动全面融入兰西城市群国家战略。城市群已成为我国新型城镇化主体形态。我们将紧抓统筹区域发展、重构区域格局的机遇，打破在自家一亩三分地上转圈圈的思维定式，主动全面融入国家战略，深化兰西城市群规划研究，落实省上实施方案。坚持兰州、西宁两极带动的要求，提升西宁在兰西城市群的极核作用、战略地位。强化“协同发展”理念，主动参与协调城市群内部产业分工协作，合力推动区域重大基础设施建设，谋划实施生态文明建设整体项目，构建更加紧密的利益共同体，打造引领区域发展的龙头，为新青海建设升级加力。

主动承担“大西宁”建设重任。建设宜居宜业“大西宁”是省委省政府赋予我们的新使命，更是我们难得的历史机遇，我们将顺应城市发展规律，以世界眼光、一流标准把握新时代西宁城市未来发展趋势。落实省委战略意图，强化兰西发展主轴，在加快推进湟中撤县设区的同时，超前规划未来向东发展方向，重构西宁在新一轮城市发展竞争中的承载骨架和发展空间。突出顶层设计、系统研究，制定“大西宁”规划建设三年行动计划，实施空间体系等专项规划，谋划建设一批“大西宁”都市圈辐射带动性重大项目，努力打造全省高质量发展的增长极、动力源。

（二）着力提升规划水平，打造高品质精致城市。对标国内外一流城市规划标准，充分发挥规划龙头作用，科学布局生产空间、生活空间、生态空间，体现战略性、提高科学性、加强协调性，描绘城市长远发展的美好蓝图。

更新城市规划理念。树立“精明增长、紧凑城市”发展策略，开展城市人口、土地、生态和安全四个维度的承载力研究，建立框定总量、限定容量的“五量控制”机制，划定“三区三线”，严控城市增长边界，牢牢守住生态保护底线。按照“一芯双城、环状组团发展”城市布局，推动城市空间与自然环境有序共生，彰显高原生态山水城市意境。实施“多规合一”，将主体功能区规划、土地利用规划、城乡规划等空间规划融合为统一的国土空间规划，推动城市总规详规、城市设计、建筑设计纵向贯通，推动空间规划、经济规划、产业规划横向融合，形成市域规划一张图。实施“城市体检”计划，认真研究“引黄济宁”背景下的“大西宁”生态山水城市格局和城市特色品牌塑造。

发挥规划引领作用。坚持规划先行、谋定而后动，紧扣提升西宁在国家战略、全省大局的战略地位，编好《“大西宁”战略规划》《西宁城市总体规划（2020—2035）》。坚持“组团发展”，严守园区片区开发边界、功能组团定位和生态隔离带范围。坚持“双城联动”，大手笔与微更新相结合，推动“城市双修”，促进城市有机更新。编制“活力轴、生活圈、风貌区”等六大标志性工程规划，塑造高品质城市空间。强化重点地段城市设计，把握重要节点特色风貌。制定乡村风貌建设指南，加强村庄风貌管控，发掘村庄特色，留住美丽乡愁。

严格规划管控。强化规划权威，改进规划审批，坚持以市规划领导小组为核心的分区域审批制，发挥专家委员会的权威性独立性，健全用途管制，监督规划实施，确保市域规划一盘棋、审批一支笔。持续推进“双增双减”，严格限定住宅项目容积率上限，严控在中心城区拆旧建新、批建高密度高层住宅，禁止沿街住宅与底商混建。建立健全多部门联动快速查处机制，清理建成区违法建设，利用腾退空间优先建设便民服务、休闲绿地、停车泊位等设施。

（三）着力推动城市扩容提质，增强综合载体服务能力。按照新片区“筑基承接”、老城区“疏解提升”的调优路径，以精品理念雕琢城市之美，以工匠精神铸造城市经典。

加快推进“一芯双城三片区”建设。坚持先地下后地上、先生态后业态，掀起重点片区生态基础设施建设新高潮。全面启动西堡绿芯森林公园建设，加速推进道路、灌溉等基础设施，建设水滩花海、碧山公园景区，造林绿化3.5万亩，区域绿化面积达到60%以上。着力推进“双城联动”，中心城区坚持疏控并举，推动适宜功能和产业向城市副中心转移，基础设施、公共服务等向三县延伸布局。多巴新城全面推进市政道路、旧村改造、湿地公园等基础设施和生态本底工程，加快多巴湖、物流园等引领性项目，形成副中心建设大场面，主动承接全省公共服务职能。加快建设南川、北川、鲁沙尔片区，南川片区以市民中心、时代大道等标志性工程为牵引，掀起会展中心、万科城等项目建设热潮。北川片区加快建设铝镁合金高新材料产业园，建成园区主要道路、标准化厂房、污水处理厂等基础设施。鲁沙尔片区全面启动民族文化旅游产业园建设，高标准编制园区总规和战略规划，开工建设药水温泉度假小镇等项目。

启动两大系统性工程。立足湟水河生态走廊，通过延伸南北渗透绿廊的宽度与厚度，优化提升沿河功能构成，展现湟水灵气秀美，促进一河两岸交汇融合，打造拥河、携河、沿河的湟水河生态活力轴。按照“做好人车路的文章”要求，启动新一轮总投资386亿元的“幸福西宁绿色交通”三年行动计划，再大干三年，为“大西宁”未来发展奠定更加坚实的交通基础。围绕便捷出行、智慧信息等八大专项行动，实施昆仑大道快速化、同仁路跨湟水河桥等项目。建成“公交都市”，开通大站大运量城市快客。再升级改造8000个停车泊位，实现电子收费全覆盖。继续调整优化轨道交通线网规划，创造条件申报争取。

加快推进公用基础设施建设。实施城市二次供水新（改）建工程，加快户表改造，建设南川加压站及管网升级等重点项目，依托“引黄济宁”开展城市南部水厂前期工作，努力实现“北水北用、南水南用”。开工建设天然气储气设施。新增地下综合管廊10公里，做好国家海绵试点城市迎检。新建人民公园等7处过街设施，人行天桥加装电梯。启动配电网高质量发展专项行动计划，三年投资100亿元、新增电量100亿千瓦时，建成国内一流、西北领先的现代化电网。

（四）着力实施“幸福西宁·高品质生活”八大工程，持续提升城市品质。我们将从群众身边的小事、烦心事抓起，继续实施好十大类30件民生实事，以优质公共服务供给顺应人民群众新期盼，确保群众安居乐业、城市安全有序、社会和谐稳定。

实施“幸福西宁·高品质生活”八大工程。启动“美丽夏都·清洁西宁”升级版，全面推行“垃圾不落地”和“全城清洗”，建立“门前六包”区域责任制，定期发布城管满意度、市容环境卫生指数，打造“最干净高原城市”。开展“花园城市”增绿提质添彩工程，调整优化公园绿地布局，提高绿化造林标准质量，增加彩叶树种和花卉配置，新增园林绿地1000亩，建成10个公园游园，营造40处花街花桥，建设9条森林徒步游彩色步道，构筑5万平方米彩色绿化空间，打造一街一品、四季常绿、花香满城的花园城市。建成市民之家，推行“集中办+智能化”服务模式，将与群众关系密切的行政审批和公共服务事项全部入驻市民中心，设立其他市州审批服务事项代办窗口，让群众进一扇门、办百项事。把社会保障、户籍办理、违章处理等高频类事项纳入移动、自助终端办理，打造“指尖上、家门口”政务服务。构建“健康服务共同体”，深化医联体改革，组建第二医疗集团，拓展服务功能，让医疗集团从单纯的“看病开药”转变为向市民提供疾病预防、医疗康复、妇幼保健、医养融合、健康管理等综合性服务，实现以治病为中心向以人民健康为中心转变，全方位全周期保障群众健康。推进“幸福养老”工程，新建50个农村老年之家，建设25个中央厨房、90个幸福食堂，打造15分钟助餐送餐配餐服务圈，实现助餐服务与医养结合、生活照料、心理慰藉等居家与社区养老服务叠加融合，托起幸福“夕阳红”。启动学前教育普惠提升工程，城镇小区严格依标配建幼儿园，已建小区经营性幼儿园逐步改造成普惠性幼儿园。提高幼儿教师待遇保障，对4500名幼儿教师和保育员予以补助。新（改）建86所城乡普惠性幼儿园，增加5500个幼儿学位，全市普惠性幼儿园占比提高到85%，有效缓解入园难、入园贵、入园远等问题。开展文体惠民工程，开工建设沈那遗址公园和市博物馆、图书馆、文化馆、美术馆，建成投运传媒中心。实施体育工作三年提升计划，提档升级“10分钟体育健身圈”，建设西门口体育健身中心和5个区级综合体育中心，推动社会场馆和中小学校场馆免费或低收费开放，滋润群众幸福生活。启动物业服务提升工程，开展“强化物业服务”三年行动，重拳整治业主大会制度不完善、物业企业服务态度差、卫生管理不到位、收费标准不统一、账务收支不透明等群众反映强烈的问题，推动物业服务管理提档升级，让服务更贴心、业主更满意。

推动社会事业繁荣发展。聚焦群众日常衣食住行，以社区为单位，建设64个“15分钟幸福生活圈”，增加社区休闲健身空间、建立宜人的步行网络体系，加强社区交往空间建设，切实激发社区空间活力，打造有归属感的现代社区。优先发展教育事业，加快消除义务教育大班额，提升中等职业教育办学能力，鼓励社会力量办学，新（改）建光华中学、文苑路小学等32所城乡学校，建成北京外国语大学西宁国际中学、湟中职教中心三期，更加重视师资队伍建设，办好人民满意的教育。探索“互联网+医疗健康”服务模式，巩固家庭医生签约服务，促进优质医疗资源下沉，开工建设市中（藏）西医康养中心，建成北川医院、市妇计中心等项目，竭力为群众提供优质医疗服务。

提高保障和改善民生水平。把稳定就业放在更加突出位置，抓好高校毕业生、农民工、退役军人、去产能分流职工等重点群体就业，城镇新增就业3.1万人，农村劳动力转移就业35万人（次）以上。下大力气解决农民工工资拖欠问题。综合改造老旧小区住房1万套、棚户区2924套和农村危旧房500所，在具备条件的多层住宅推广加装电梯。落实收入提升行动，继续提高退休人员基本养老金标准，健全最低工资标准增长和国有企业工资决定机制。有序推进全民参保计划，健全职业年金、企业年金、商业保险等多层次养老保险制度体系。严控蔬菜批零差率，加强储备调运，扩大平价蔬菜店覆盖面和普惠度，实行节日期间直补限价，确保物价总体平稳。做好城镇贫困和困难群体精准帮扶工作，完善社会救助、社会福利、慈善事业、优抚安置等制度，健全农村留守儿童和妇女关爱服务体系，稳步提高残疾人社会保障服务水平，把温暖送到群众心坎上！

推进城市精细化智能化管理。建设数字城市、智慧城市，完善城市运行指挥中心应急指挥、城市管理、便民服务、辅助决策功能，做强“城市大脑”，把感知触角延伸到与群众生活、城市运行紧密相关的各个领域。加快建设“信用西宁”，坚决惩治各类失信“老赖”。深化城管体制改革，用好街长管家力量，建立“街道吹哨、部门报到”一街一策机制。注重城市品牌创建。大力弘扬新青海精神和西宁精神，以独特文化印记塑造城市“灵魂”和“气质”，以丰满的精神滋养城市。

加强和创新社会治理。继续巩固民族团结进步创建成果，深化“平安寺院”建设，依法加强宗教事务管理。坚持底线思维，加快建设“平安西宁”，深化扫黑除恶专项斗争，坚决打击“三股势力”。创建国家安全发展示范城市，健全应急管理体系，牢牢守住城市安全和社会稳定底线。坚持党建引领激发社区活力，提高社区治理能力。推进国家食品安全示范城市创建，打造“绿色餐厅”，推广“明厨亮灶”分类定级评价和食用农产品二维码追溯管理，让群众吃得放心。发挥信访联席会议机制作用，推进信访矛盾化解攻坚。同时，做好全国第四次经济普查，启动“十四五”规划编制，推进“七五”普法，加强退役军人管理服务，深入开展全民国防教育、双拥优抚，支持工会、共青团、妇联等人民团体发挥桥梁纽带作用，继续抓好人防、地震、气象、档案、地方志等工作。

各位代表，宜居宜业“大西宁”是我们共同生活的美好家园，我们要把城市当成自己的家一样去呵护、去热爱、去建设，汇聚各族干部群众的智慧和力量，共建共享新时代幸福西宁，让每个西宁人都能从城市发展中分享更多成果，在城市生活中更加幸福！

各位代表，新形势、新任务对各级政府提出了新的更高要求。我们将加强政治建设，牢固树立“四个意识”，不断坚定“四个自信”，把坚决做到“两个维护”作为根本政治任务，以“两个绝对”为标准，以“两个绝对”具体化为载体，加强斗争历练，增强斗争本领，永葆斗争精神，始终做习近平新时代中国特色社会主义思想的坚定信仰者和忠诚实践者。我们将加强法治建设，始终把依法行政贯穿于政府工作全过程，自觉接受市人大、市政协及各方监督，全面推进政务公开，有效畅通舆论监督和社会监督渠道，努力建设人民满意的法治政府。我们将加强廉政建设，深入推进党风廉政建设和反腐败斗争，严格执行中央八项规定及其实施细则精神和省市若干措施，带头过“紧日子”，压减一般性支出，严控“三公”经费预算，强化干部廉政教育和管理，严惩发生在群众身边的腐败，努力建设人民满意的廉洁政府。我们将加强作风建设，开展“基层减负年”活动，集中整治形式主义、官僚主义突出问题，防止“文山会海”，大力压缩规范各种督查检查考核，让基层有更多精力抓落实谋发展；开展“政策落实年”活动，强化政务督查、履职问责，勇于担当作为，善于攻坚克难，不断提升行政效能和服务水平，努力打造人民满意的服务政府。

各位代表，蓝图绘就新篇章，梦想铺筑幸福路。我们将更加紧密地团结在以习近平同志为核心的党中央周围，在省委省政府和市委的坚强领导下，紧紧依靠全市各族人民，凝心聚力、开拓进取，以打造绿色发展样板城市、建设新时代幸福西宁的优异成绩，交出一份无愧于时代、无愧于人民的合格答卷，向中华人民共和国成立70周年和西宁解放70周年献礼！
